# Joan Heath interviews Suzanne Cory and Joan Steitz: a female perspective of science in the swinging ‘60s

**DOI:** 10.1242/dmm.049609

**Published:** 2022-06-06

**Authors:** Joan Heath, Suzanne Cory, Joan Steitz

## Abstract

Prompted by the occasion of International Women's Day, Joan Heath and DMM reunited Professors Suzanne Cory and Joan Steitz via Zoom to discuss their extraordinary careers and joint experiences in science. They also delve into past and present challenges for women in science, and discuss the role of scientists in a post-pandemic world.


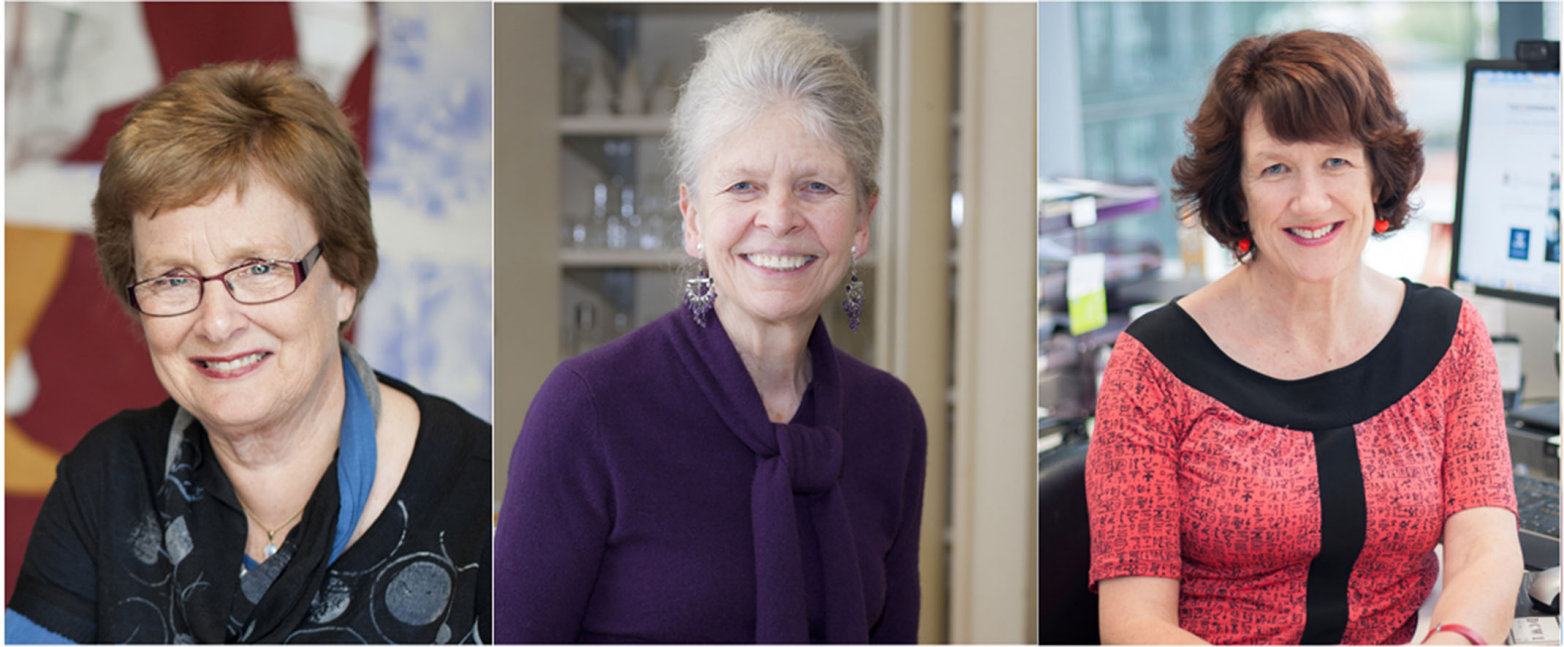


**Suzanne Cory, Joan Steitz and Joan Heath (from left to right)**

As one of Australia's most eminent molecular biologists, with a school in Melbourne bearing her name, Professor Suzanne Cory has been both Director of The Walter and Eliza Hall Institute of Medical Research in Australia (WEHI) and President of the Australian Academy of Science. She earned her PhD at the Medical Research Council (MRC) Laboratory of Molecular Biology (LMB) in Cambridge, UK, with postdoctoral training at the University of Geneva. She continues her research at WEHI as an honorary distinguished research fellow, investigating the genetics of the immune system in the development of blood cancers and the effects of chemotherapeutic drugs on cancer cells.

Joan Steitz – currently Sterling Professor of Molecular Biophysics and Biochemistry at Yale University, and for 35 years the recipient of a Howard Hughes fellowship – is best known for her seminal work in RNA biology. She was the first female graduate student to join the laboratory of James Watson at Harvard University and proceeded with her postdoctoral training at the MRC LMB in Cambridge. Her pioneering research delved into the fundamental mechanisms of ribosome and messenger RNA interactions, as well as RNA splicing, heralding the phenomenon of alternative RNA splicing. A recipient of many awards and honours, she is also involved in international projects aimed at supporting women in science.

Host Joan Heath heads a laboratory at WEHI in Australia. She received her undergraduate degree from the University of Cambridge, followed by her PhD at the Strangeways Research Laboratory also in Cambridge, then just across the road from the MRC LMB. After postdoctoral positions in bone biology and osteoporosis research, Joan joined the Ludwig Institute for Cancer Research where she became a laboratory head, and changed her focus to cancer research using zebrafish to identify genes that are indispensable for the rapid growth and proliferation of cells during development. She joined the WEHI in 2012. There she showed that the same developmental genes are also required by highly proliferative, difficult-to-treat cancers, including lung, liver and stomach cancer, paving the way for translational research targeting these genes in novel cancer therapies.

**Joan H: How long have you two known each other?**

**Suzanne:** I was calculating that this morning and I was astonished because it seems like only yesterday, but it has been 55 years since we met in Cambridge. It has been a voyage in science and a voyage in the world because we have always made a point to meet up in beautiful places and go hiking. That is how we've been able to renew our friendship over all these years.

**Joan H: Where were you when you first met?**

**Joan S:** We both were working at the MRC LMB in Cambridge, England. Suzanne was doing her PhD and I arrived slightly later for a postdoc.

**Suzanne:** We had a pre-meeting in the sense that Joan, Jerry Adams (my future husband) and Tom Steitz (Joan's husband), were all graduate students together in Harvard. So, when Joan and Tom came to Cambridge, it was natural that we would all start doing things together. And Joan and I ended up sharing a lab bench.

**Joan S:** The reason that I did a postdoc in the mecca of X-ray crystallography was that I had married a crystallographer – and there was no other place that he could possibly go. They very much wanted to have my husband at the Cambridge MRC lab, but there wasn't a clear plan for me. Francis Crick suggested that I do a literature project in the library, but I knew that theory was not my forte in comparison to experiments. I started talking to the many people working in the lab and found a project that no one wanted, because it was so challenging. But it was a very interesting problem, so I decided to take it on – and it turned out to be a great project.

**Joan H:** That's amazing. You were obviously determined to overturn other people's expectations of you.

Suzanne, even now, it's extremely unusual for a young person to leave their home country to do their PhD. It's still a brave thing to do but all those years ago it was really courageous. You told me that you ended up there because you wrote a simple letter, which was a complete shot in the dark.

**Suzanne:** It certainly was. During my master's degree at the University of Melbourne, I became more and more interested in doing science and decided I would do a PhD. But I had a counteracting desire to travel and see Europe. So I decided that I would do my PhD overseas to give myself the opportunity of travelling. I had fallen in love with DNA during my undergraduate studies. So, I wrote a letter to Francis Crick in Cambridge, and asked if he would take me on as a PhD student. Much to my amazement, I eventually got a letter back saying yes. I think that my professor of biochemistry might have also visited Cambridge while he was travelling and spoken up for me. However, I was still extraordinarily fortunate that Francis had agreed because there weren't many PhD students in the LMB at that time. It made such a difference to my entire life. I look back on that letter and think, “How did you have the audacity to write that letter and aim to go to that laboratory?”. I think it was partly naivety.

**Joan H:** That's a lesson for everyone, to go for your dreams, and don't assume people won't take notice of you. It is more difficult now, when scientists receive hundreds of e-mail applications from prospective PhD students in their inbox. You would have written a letter with a stamp on it that probably took three weeks to arrive, but it just shows you that you should be audacious. Did you have a different experience to Joan when you arrived? **Was there a proper project already lined up for you?**

**Suzanne:** I was interviewed by Francis Crick and Sydney Brenner, who were the joint directors of the department. They decided that I would work on the structure of the methionyl-tRNA that puts methionine into internal positions in polypeptides. After they described the project – which involved doing counter-current distribution fractionation of bulk tRNAs, in which I had no experience whatsoever – Sydney in his very characteristic monotone said, “Do you think you're up to it?”. I sort of gulped to myself and said, “Yes, I think I could do that”. I then went to Brian Clark's laboratory, who was going to be my PhD supervisor, and started the project. Like always in life, if you learn from people and just go from one day to the next, you actually get there in the end.

**Joan H:** So, persistence was key. **Were there many other women at the LMB at the time?**

**Suzanne:** I don't remember any female scientists who had official senior positions. There were certainly some strong female scientists there, but I don't think they were given the recognition or the status that they actually deserved.

**Joan S:** Later, some were given more recognition, crystallographers in particular, but not so much the molecular biologists.

**Suzanne:** I think, as women, we both pioneered in that department.

**Joan H:** Given the fact that you both agreed to take on projects you had very little previous experience with and that the male supervisors thought you weren't going to have the mettle to carry it through, **once you were there**, **did you feel that you had to work the whole time? Or did you still manage to have lots of fun and partake in opportunities that Cambridge had to offer at the time?**

**Joan S:** We certainly partook in a lot of those things. My husband and I got interested in antique furniture, antique paintings, and used to scour the countryside for little antique shops. We saw lots of England, then a little bit of Scotland and Wales. It was wonderful. A real adventure.

**Suzanne:** I worked really hard most of the time that I was in Cambridge, as the work was very exciting. But I would take holiday periods, camping and youth hostelling all over Europe with a girlfriend from Melbourne and later, travelling with Jerry. We also would go to London for the opera and looking for amazing clothes on Carnaby Street and Chelsea Road (this was the Beatles era, late 60s). Jerry once came back with a purple velvet suit, which was his prized possession for many years. There was lots of fun but also lots of work.

**Figure:**
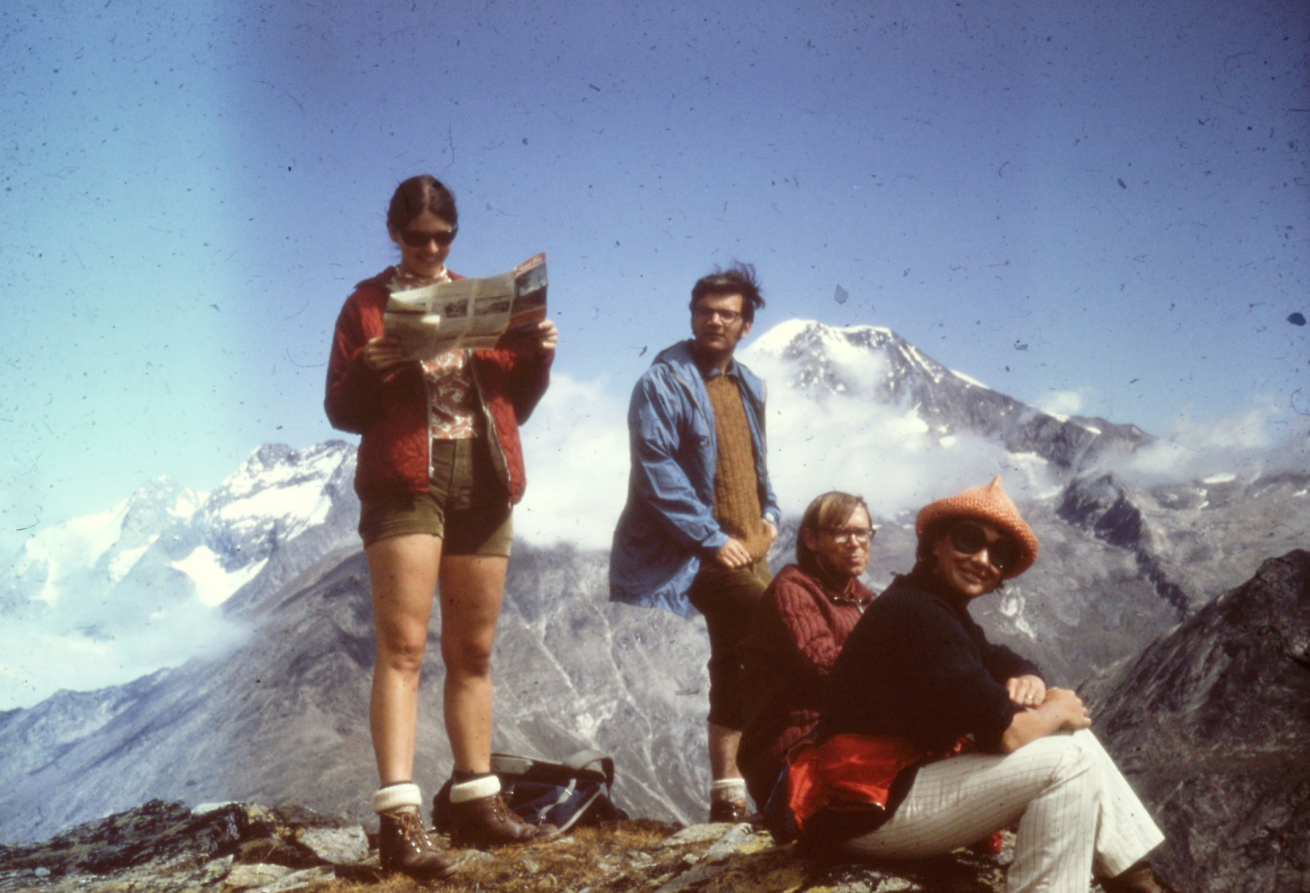
**Joan Steitz, Tom Steitz, Jerry Adams and Suzanne Cory (from left to right) in the Swiss Alps, 1970.** Image courtesy of Mark Bretscher. This image in not reproduced under the terms of the Creative Commons Attribution 2.0 Generic license. For permission to reproduce, contact the DMM Editorial office.

**Joan H: Can you remember the first moment in that part of your career that gave you the most pleasure?**

**Joan S:** I worked on a project for about a year, and it turned out that I was doing the wrong fractionation method to get the material that I needed to analyse. Then I had a conversation with Sydney Brenner telling him that I was going to give this one more try with a new method, and then I was going to give up. I remember Sydney saying, “Sometimes, like with a bad marriage, you have to give experiments one last try before you give them up.” Then I tried again, and it worked. This is often the case in science, that you try something new, that's a little bit different, and it makes all the difference. Then you're running.

**Suzanne:** The same thing happened to me. I was labouring away on the counter current distribution machines fractionating methionine tRNA, with the goal of sequencing it by the laborious procedure recently published by Robert Holley. However, Fred Sanger, in the department upstairs, had invented a totally new method for sequencing using ^32^P-labelled RNA. I desperately wanted to try this, so I managed to persuade my supervisor that we should change techniques. That change was key to my future because the approach was successful. I still remember to this day exactly where I was in Cambridge, walking on a Sunday afternoon, when the last piece of the puzzle dropped into place in my mind, and I had the entire sequence. In that moment, I was extremely joyful, because I knew I had my PhD and that I had succeeded. So that was my eureka moment.

**Joan H:** Obviously, these were extremely productive years, and you've mentioned several Nobel Prize winners in your midst. It must have been the most inspiring environment, which I'm sure had a big impact on what you did next. **By this stage in your career, were you already feeling ambitious or was it still your scientific curiosity that was driving your path?**

“I expected that I would go back to the United States and be a research associate in some man's lab […]. Then it turned out that people were more impressed than I thought and started offering me junior faculty jobs.”

**Joan S:** I had gotten a lot of recognition for having sequenced a piece of mRNA, using the same methods that Suzanne used to sequence tRNA. However, I had no expectations, because I had never seen a woman as a science professor, or head of a lab. I expected that I would go back to the United States and be a research associate in some man's lab, and maybe they'd let me guide a graduate student. Then it turned out that people were more impressed than I thought and started offering me junior faculty jobs.

My husband had already secured a junior faculty job in Berkeley before we even went to England, so we went back there after two years. My husband went to the chair of the department in Berkeley and put down letters on his desk of job offers that both of us had received for independent, junior faculty positions from several universities. The Chairman then said to Tom, “But all of our wives are research associates in our labs, and they love it”. This tore at my pride, as there had been a couple of universities that offered us both faculty jobs, and Berkeley was only offering one. So, we didn't stay at Berkeley, and we came to Yale, which was wonderful.

**Suzanne:** It's really amazing to think that they gave you up. How foolish they were.

**Joan H:** They've lived to regret it a million times over. **Suzanne, at that point were you ready to climb this very difficult ladder?**

**Suzanne:** Like Joan, I didn't have any expectations. For me, it was a matter of being able to continue discovering things in science. Jerry had already arranged to start a postdoc in Geneva. So, I applied for a postdoctoral fellowship, and obtained one. We went off together to Geneva to start our married life, and that was the beginning of us doing science together, which we've done ever since. I think without Jerry guiding me at that stage in my life, I would have probably drifted out of science. I don't think I had the scientific confidence to ever think that I would be running a lab. For me, it was just continuing a voyage of discovery; and being lucky to end up in a wonderful scientific partnership and, through that partnership, my confidence grew over the years.

**Joan H: How many years after your postdoctoral training was it before you looked around your environment and had the confidence to think that you could be a lab or department head or could run an Institute?**

**Joan S:** I would say that confidence just grew. Tom and I were part of a departmental overhaul that involved hiring about six new people at Yale. We all stuck together, supported each other and were very collegial even though we worked in different areas. I think the collegial nature of the department in Yale helped me gain confidence. It was very scary at first because I didn't know if I could write grants or direct people.

**Suzanne:** Cambridge had an incredible influence, certainly over me, and I'm sure over Joan, Tom and Jerry, too. We looked around and saw all these amazing Nobel laureates, but also all these very ambitious, talented postdocs from around the world. I don't think anyone thought about being the head of a department at that stage. We were simply striving to make discoveries and we gave each other mutual confidence, and stiff competition, too.

The other thing that Cambridge gave us, was a new technology. For Joan and me, it was RNA sequencing. Being able to do that technology opened doors all around the world. I now always advise young people to go to the best place in the world to train in your chosen subject and acquire a new technology, because that will open the door to many opportunities in the future.

Jerry and I made some excellent discoveries in Geneva, which were published in front-rank journals. Then it was time to move to full independence. I really wanted to go back to Australia but, as Jerry is an American, it was not at all obvious that he should take the big leap of moving to the bottom of the world and starting a lab there. I owe him a tremendous debt because he decided that he would take that risk.

Earlier, whilst on our honeymoon, we had visited various labs in Australia. Although WEHI was an institute for immunology, a field we knew little about at that stage, it had the same atmosphere as the LMB in the sense that everyone was striving at the frontiers of science and competing with the rest of the world. We decided this was the only place in Australia that we would work at and that we would attempt to persuade the new director Gus Nossal that he needed molecular biologists. We had an interview with him in Switzerland and he offered us jobs as postdocs. Again, we were probably very naive and audacious but we told him we didn't want to be postdocs – we wanted to run our own lab. And he agreed and we launched our fledgling lab together in 1971. What drove us was always discovery, rather than career ambitions.

**Joan H:** You've both described these amazing sets of circumstances that were challenging but, nevertheless, very satisfying. However, a lot of things have since changed. **What do you think are the main remaining barriers to women in science?**

**Joan S:** There is an important phenomenon called social identity threat, or stereotype threat, that I think still impedes women in proceeding in their careers. The phenomenon is described by cognitive psychologists as a reaction that all people experience if they feel that they are part of an undervalued minority. And so, by definition, since there are fewer women in science than there are men, women are being subjected to stereotype threat. Cognitive psychologists have studied the physiological manifestations of this, including increased heart rate and perspiration but, psychologically, they've also documented that cognitive learning and memory are impaired when one has these feelings.

I first learned about this in 2007 and I looked back and realized why, for 30 years, when I'd been on committees as the only woman amongst ten men, I wouldn't dare say anything – because I was frightened stiff. Men undergo this response, too, if they're put into the situation of being undervalued. If you understand why you're reacting the way you're reacting and know that this is a normal human response, I think it helps you to overcome your own feelings of insecurity and allows you to go ahead. I always tell young women who I'm rooting for in science about this, because I want them to know that they will very likely end up feeling this way, and it's a normal human response.

“One thing I sometimes get frustrated about is that we often need men to change things […] but what we really need are women in those high-level positions, so that they can be the champions of change.”

**Joan H:** There are other terms describing other relevant phenomena, such as unconscious bias, imposter syndrome and champions of change. One thing I really relate to is imposter syndrome. I've listened to webinars on this topic and they all reach a similar conclusion that we all feel the same. On the one hand, at the end of the webinar, you do feel somewhat elated to know that it's not just you, and that it's normal. But, on the other hand, it doesn't really change things. It's a recognition of what we feel, and we all get some help from that, but you really need opportunities to change things at a higher level. One thing I sometimes get frustrated about is that we often need men to change things, leading to this concept of male champions of change. I admire those men; but what we really need are women in those high-level positions, so that they can be the champions of change. Not having 50% of university departments and medical research institutes run by women still limits our full potential.

**Joan S:** I certainly agree with you, Joan. It's very important to have realistic role models. Suzanne being head of the WEHI for all those years has engendered all sorts of admiration.

**Joan H:** During that period, Suzanne not only did fantastic science but our Institute doubled in size.

It's transformative when you have women making up 50% of people around the table. It's no help just having a token female because that poor person's not going to be able to change everything on her own. **In American scientific institutions, are there any firm quotas for female scientists and other people that are underrepresented in science?**

**Joan S:** In recent years there has been a push in that direction. Sometimes it's successful and sometimes it's not. It is very different now compared to when there was no consciousness that this was unfair or that things could be better if we had real representation.

**Suzanne:** I agree with both of you in everything that's been said. While reflecting at this moment, what it says to me is that what's really needed is societal change, and that we need to give courage to girls from the very earliest age. It should come naturally, they shouldn't feel inferior, and others should not look at them as inferior. They should expect to have careers as well as families, be able to manage both and have somebody alongside them who helps them manage both.

I think that affirmative action for women in science is necessary because the pace of change has been so slow. However, I also think quotas can be detrimental to the cause of women, in the sense that it's then possible for people to say you only made it because there was a quota – which is very destructive. If I look back on our careers in science, it is clear that things have changed tremendously. Today there are more opportunities for women because many universities and institutes are bending over backwards to equalise things. The downside of this is that talented men may miss out on positions because of this policy and the pendulum could swing back.

**Joan H:** The evidence shows that when more women are involved in things, those things go better. For instance, boards that have more women on them are more productive. Obviously, what you alluded to is there are lots of fantastic male scientists as well. The real issue here is there's not enough funding to go round to support all the great men and women. But there are clearly enough good women around to be represented at the 50% level, without disproportionately disadvantaging male scientists.

**Joan S:** Men and women are now operating on a more even playing field, which doesn't mean that the men are missing out. They're just in a more-competitive situation – as they should be.

**Joan H: Suzanne previously covered the specific advice she would give to young female researchers. Joan, do you have any other suggestions?**

**Joan S:** I encourage them to try lots of different things in science, and when they find something that really grabs them, then go for it and be persistent. We all know that science is very up and down. But if you keep pushing when you're in a trough, it will always go back up again and you will succeed. That's harder for a young person, who hasn't experienced these troughs, to understand.

**Joan H:** Yes, and the period when women scientists start having children is the hardest part. It's still a choice that some women make, to take some years off and come back with a less ambitious plan for their career. Obviously, things like maternity leave payments and so on are improving but there's no question that, in most circumstances, the research will slow down during that period.

**Suzanne:** What I say to young women at that stage of their careers is that you have to be very focused, you must spend the time that you do have in a very focused manner, so that you can be the most productive you can be. But you have to be supported at home by your partner. If you're both scientists it's easier because you can appreciate why the other person is rushing into the lab late at night, for example, but for most people, that's not true. So, what is really important is equal sharing of responsibilities from both partners when young families are around. And I think employers need to give both of those partners a longer time to achieve the kind of papers that they need to progress in their careers. That's a period when it is much harder to be productive, and we need to continue to support people during that difficult phase of their careers because we've invested so much in them. They have so much to offer to science and to society, so to let them slip out at that stage is a great waste.

**Joan H:** Let's change tack a little bit and think about some of the broader challenges in science. **What do you think the COVID-19 pandemic has taught us about the importance of clear scientific communication and real engagement with the community?**

**Joan S:** Whenever I talk to people about this, I very clearly make the point that it was decades of fundamental research that led to the development of the COVID-19 vaccine. If it hadn't been for those fundamental discoveries in how cells and mRNA work, it would never have only taken 63 days from sequencing the virus to phase one clinical trials at Moderna. I try to point out to people that all the different discoveries coming in from different angles made that possible. I personally find it absolutely remarkable that all that knowledge could be harnessed, so very quickly. I've been doing fundamental research my entire life and I never expected to see it materialise in the way it has. It's a wonderful reward.

**Joan H: Do you think this has resulted in the community appreciating scientists more?**

**Joan S:** I don't think we're far enough downstream to know that. In the US, there has been a congressional vote to abandon our maintenance of vigilance and preparedness for future pandemics – which seems ridiculous. Now we have all these procedures set up, all we have to do is maintain them for the next one. Whereas, if we just let go of these procedures, we'll have to start over again for future pandemics. I guess we're not good enough at communicating some of these things at this point.

**Joan H:** Millions of people died from the virus and yet, if we hadn't had the vaccines, the scale would have been even more horrific. If we were able to convey this information effectively to the public, then, hopefully, people would recognise that – as well as spending a fixed percentage of the gross domestic product on defence, for example – we should spend at least the same amount on science. Not only for pandemics but for tackling climate change and other pressing issues. I like to think this is an auspicious time but I don't know whether we are really taking advantage of it.

**Suzanne:** The pandemic has brought science and scientists to the forefront, and there has been a period of great respect for scientists having developed the vaccine. It's an absolute miracle that it was done so fast and effectively. We're very fortunate but, as Joan said, that was not luck. It was through investment in basic science for decades. We have to keep conveying this message, to our politicians in particular, so that they will keep supporting all kinds of scientists, because we never know what's around the corner.

**Joan H:** Certainly, people like Anthony Fauci in the US and Catherine Bennett in Melbourne, spoke eloquently and had a real talent for communicating things clearly and in a nutshell. That's not something we're all good at and it's not something that is easy to train into people either. I think we all need to try to capture the attention of the community at large, by speaking plainly. I don't think people understand that scientists are underfunded and could do so much more if funding was more generous.

“All I can say to young people is, if you really love science and have a passion for it, keep trying – because you will succeed if you put your whole heart and soul into this career path.”

**Suzanne:** I think the general public has no appreciation of how tenuous the life of a scientist can be, and how we are losing so many great minds entering the field because young people just finishing their PhDs look with dismay at how hard it is to support a career in science and get enough funding. There's a tremendous waste of talent. All I can say to young people is, if you really love science and have a passion for it, keep trying – because you will succeed if you put your whole heart and soul into this career path.

**Joan H:** This has been an absolutely fantastic discussion and it's such a delight to talk to women who, after all these years, are still as passionate as ever and are pursuing their scientific subjects with the same vigour as they have all along.

**Suzanne:** It's been wonderful to talk with you, Joan, and I hope that we see each other soon, no matter what continent. And thank you, Joan Heath for getting us together and giving us this opportunity.

